# Assessment of drug therapy problems among patients with cervical cancer at Kenyatta National Hospital, Kenya

**DOI:** 10.1186/s40661-017-0054-9

**Published:** 2017-10-18

**Authors:** Amsalu Degu, Peter Njogu, Irene Weru, Peter Karimi

**Affiliations:** 1 Department of Pharmaceutics and Pharmacy Practice, University of Nairobi, College of Health Sciences, School of Pharmacy, P.O. Box 19676-00202, Nairobi, Kenya; 2 Department of Pharmaceutical Chemistry, University of Nairobi, College of Health Sciences, School of Pharmacy, Nairobi, 19676-00202 Kenya; 3 Kenyatta National Hospital, Division of Pharmacy, Nairobi, 20723-00202 Kenya

**Keywords:** Drug related problems, Cervical cancer, Kenyatta national hospital

## Abstract

**Background:**

Although cervical cancer is preventable, it is still the second leading cause of cancer deaths among women in the world. Further, it is estimated that around 5–10% of hospital admissions are due to drug related problems (DRPs), of which 50% are avoidable. In cancer therapy, there is an immense potential for DRPs due to the high toxicity of most chemotherapeutic regimens. Hence, this study sought to assess DRPs among patients with cervical cancer at Kenyatta National Hospital (KNH).

**Methods:**

A cross-sectional study was conducted at the oncology units of KNH. A total of 81 study participants were recruited through simple random sampling. Data were collected from medical records and interviewing patients. The appropriateness of medical therapy was evaluated by comparing with National Compressive Cancer Network and European Society for Medical Oncology practice guideline of cervical cancer treatment protocol. The degree of adherence was determined using eight-item Morisky medication adherence scale. The likelihood of drug interaction was assessed using Medscape, Micromedex and Epocrates drug interaction checkers. The data were entered in Microsoft Excel and analysed using statistical software STATA version 13.0. Descriptive statistics such as mean, percent and frequency were used to summarise patients’ characteristics. Univariable and multivariable binary logistic regression were used to investigate the potential predictors of DRPs.

**Result:**

A total of 215 DRPs were identified from 76 patients, translating to a prevalence of 93.8% and a mean of 2.65 ± 1.22 DRPs. The predominant proportion of DRPs (48.2%) was identified in patients who had been treated with chemoradiation regimens. Adverse drug reactions 56(69.1%) and drug interactions 38(46.9%) were the most prevalent DRPs. Majority (67.9%) of the study population were adherent to their treatment regimens. Forgetfulness 18(69.2%), expensive medications 4(15.4%) and side effects of medications 4(15.4%) were the main reasons for medication non-adherence. Patients with advanced stage cervical cancer were 15.4 times (AOR = 15.4, 95% CI = 1.3–185.87, *p* = 0.031) more likely to have DRPs as compared to patients with early stage disease.

**Conclusion:**

Adverse drug reactions, drug interactions, and need of additional drug therapy were the most common DRPs identified among cervical cancer patients. Advanced stage cervical cancer was the only predictor of DRPs.

## Background

In the past few decades, medicines have had a substantial positive effect on health by reducing mortality and disease burden. Interestingly, there is ample evidence that potential problems exists since the right medicine does not always reach the right patient and around 50% of all patients fail to take their medication correctly [1]. Moreover, irrational use of drugs is a major global problem, and World Health Organization (WHO) estimates that above 50% of all drugs are prescribed and dispensed inappropriately with consequent wastage of scarce resources and widespread health hazards [2].

A drug-related problem (DRP) is defined as an event involving drug therapy that has a potential to interfere with the desired health outcomes [3]. Alternatively, a drug therapy problem is any detrimental event experienced by a patient which impedes attainment of the desired goals of treatment. In the absence of appropriate intervention, medication problems have considerable negative impact on the health of the patients [4].

Drug-related problems are categorised into different classes, namely need for additional drug therapy, medication use without indication, improper drug selection, overdosage, sub-therapeutic dosage, adverse drug reactions (ADRs), drug interactions, inappropriate laboratory monitoring and non-adherence [4].

In cancer therapy, there is a tremendous potential for DRPs due to the high toxicity and the complexity of most chemotherapeutic regimens [5]. Cancer patients have a high incidence of coexisting chronic diseases and the treatment of cancer carry an inherent risk of DRPs [6]. Moreover, problems arising due to drugs are more common in cancer patients, and commonly present a major hurdle to health care providers [7].

Drug-related problems due to cancer chemotherapy can have severe consequences arising from the high toxicity and narrow therapeutic range of anticancer drugs [5]. Anticancer agents are differentiated from other class of drugs due to the frequency and severity of side effects at therapeutic doses [8]. Chemoradiation with cisplatin is associated with increased acute haematological and gastrointestinal toxicity in cervical cancer patients [9]. Since cancer patients receive multiple drug therapy, they are at a higher risk to develop DRPs. Accordingly, a substantial clinical need is required to address this problem by identifying cancer therapy-induced problems. Moreover, the prevalence of DRPs in patients with cervical cancer is not known in Kenya though the chemotherapeutic agents are expected to produce serious adverse outcomes to the patients. Thus, it was imperative that assessment was carried out to identify DRPs in cervical cancer patients to overcome these hurdles.

An extensive study of DRPs would render valuable perspicacity for the healthcare providers to lessen the incidence of DRPs [10]. However, there is a paucity of data on comprehensive DRPs among cervical cancer patients. Therefore, this study investigated the prevalence, types and predictors of DRPs in cervical cancer patients admitted at the oncology units of Kenyatta National Hospital.

## Methods

### Study design and setting

A cross-sectional study design was conducted from April to June 2017 at the oncology units of Kenyatta National Hospital (KNH), the biggest tertiary hospital in Kenya. Single population proportion formula was used to calculate the sample size [11].

$$ \mathbf{n}=\frac{{\mathbf{Z}}_{\frac{\kern0.75em \boldsymbol{\upalpha}}{2}}^{\kern1em 2\kern0.75em }\mathbf{x}\ \mathbf{P}\left(1-\mathbf{P}\right)\ }{{\mathbf{d}}^2} $$

where: n is the minimum sample size required for large population (≥10,000).

*Z*_*α*/2_ is the critical value for a 95% confidence interval (= 1.96 from Z- table).

P is the proportion of drug-related problems in cervical cancer patients. Since there were no previous studies in Kenya, P was assumed to be 50% (0.5).

d is the margin of error (5%)

$$ \mathrm{Hence},\mathrm{estimated}\  \mathrm{minimum}\  \mathrm{sample}\  \mathrm{size}\ \left(\mathrm{n}\right)=\kern0.5em \frac{(1.96)^2\kern0.5em \times 0.5\left(1-0.5\right)}{(0.05)^2}=384 $$

However, since study population was less than 10,000, we estimated the sample size using the following reduction formula.

Corrected sample size $=\frac{\mathbf{n}\ \mathbf{x}\ \mathbf{N}}{\mathbf{n}+\mathbf{N}}$ Where N = source population and n = estimated sample size for *N* ≥ 10,000 population. According to KNH Health Information Department report, an average of 90 cervical cancer patients was on treatment in both inpatient and outpatient oncology units of KNH in the preceding three months period (September–November, 2016). The study was carried out for three months period, and hence the approximate size of the source population was 90 cervical cancer patients. Then, corrected sample size =$\frac{384\ \mathrm{x}\ 90}{384+90}=73$. Therefore, the corrected sample size with a 10% contingency for incomplete medical records of the patient and non-response provided a final sample size of 81 cervical cancer patients.

### Eligibility criteria

Patients aged 18 years and above with documented diagnosis of cancer and, treatment regimens were targeted. However, only those who signed the informed consent were included in the study.

### Data collection techniques

Two qualified nurses from the oncology units of KNH were trained to assist in data collection. Relevant information about each patient such as socio-demographic characteristics, histological types of cervical cancer, stage of cancer, types of co-morbidity, treatment regimen, ADR, the rate of adherence and reasons for non-adherence, were recorded by reviewing medical records and interviewing the patients. A pilot study was done in 10% of the sample size to ensure the validity of the data collection instruments. After pre-testing, all necessary adjustments were executed on the data collection instruments before implementing in the main study. The adequacy of medical therapy was evaluated using National Guidelines for Cancer Management in Kenya [12], National Compressive Cancer Network (NCCN) practice guideline of cervical cancer treatment [13], European Society for Medical Oncology (ESMO) practice guideline of cervical cancer [14] and WHO cancer pain management protocols [15]. The probability of drug interaction was assessed using Medscape, Micromedex, Web MD and Epocrates drug interaction checkers. The degree of adherence was determined using Eight-Item Morisky Medication Adherence Scale [16]. The Modification of Diet in Renal Disease (MDRD) Study eq. [17], Du Bois method [18] and Calvert formula [19] were used to determine estimated Glomerular filtration rate (eGFR), body surface area and carboplatin dosing, respectively. DRPs were categorised as the need of additional drug therapy, medication use without indication, improper drug selection, overdosage, sub-therapeutic dosage, adverse drug reactions, drug interactions, inappropriate laboratory monitoring and patient’s non-adherence by the Cipolle et al. classification system [4].

### Analysis

The data were entered into the Microsoft Excel worksheet and analysed using statistical software STATA version 13.0. Descriptive statistics such as percent and frequency were used to summarise categorical variables of patients’ characteristics. Mean and standard deviation were used to compile continuous variables. The univariable and multivariable binary logistic regression analyses were employed to investigate the potential predictors of DRPs. A *p*-value of ≤0.05 was considered statistically significant.

## Results

### Sociodemographic characteristics of study participants

The study was conducted among 81 cervical cancer patients. The mean age of the study population was 53.3 ± 11.6 years, and the predominant portion of the study subjects 47(58.0%) were aged 50 years and above. Among the 81 study participants, 61(75.3%) were married, 44(54.3%) had a primary level of education, while only 2(2.5%) had attained tertiary level of education. Twenty four participants (29.6%) were housewives. The monthly income level of majority of the population 59(72.8%) was less than USD 100, and most of the patients 40(49.4%) were on treatment with 5–9 drugs (Table 1).

**Table 1:** Sociodemographic characteristics of the study participants

Variables	Frequency	Percent
Age (years)		
29–39	10	12.3
40–50	24	29.6
≥ 51	47	58.0
Marital status		
Single	20	24.7
Married	61	75.3
Level of education		
Illiterate	10	12.3
Primary	44	54.3
Secondary	25	30.9
Tertiary	2	2.5
Occupation		
Housewife	24	29.6
Retired	9	11.1
Merchant	5	6.2
Unemployed	19	23.5
Farmer	16	19.8
Daily labourer	4	4.9
Private employee	3	3.7
Other	1	1.2
Monthly family income (USD)		
Very low (<100)	59	72.8
Low (100–200)	18	22.2
Average (200–500)	4	4.9
Number of drugs per patient		
< 5	31	38.3
5–9	40	49.4
> =10	10	12.3

### Clinical characteristics of the study participants

**Fig. 1:**
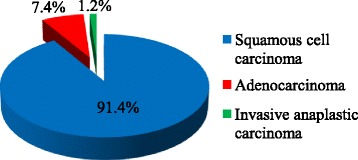
Histological types of cervical cancer among the study participants

As illustrated in Fig. 1, three histological types of cervical cancer were identified among the study subjects. Squamous cell carcinoma (91.4%) was the most common type, followed by adenocarcinoma (7.4%) while invasive anaplastic carcinoma (1.2%) was the least common histological type.

**Fig. 2:**
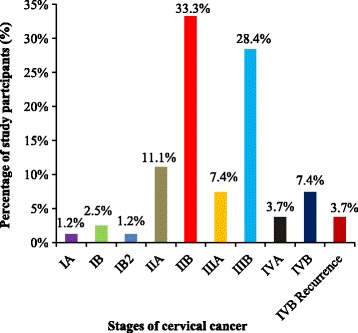
Stages of cervical cancer identified among study participants

The study showed that 44.4% and 35.8% of study population had stage II and III cervical cancer, respectively, with stages IIB (33.3%) and IIIB (28.4%) being the most prevalent. However, stages I and IV had low prevalence rates (Fig. 2).

**Fig. 3:**
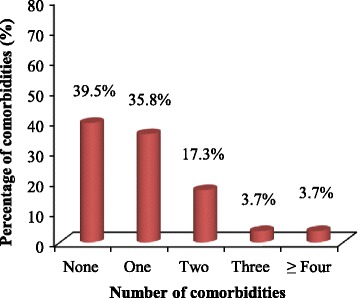
Percentage of co-morbidities among patients with cervical cancer

**Fig. 4:**
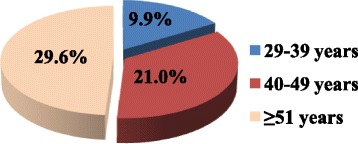
Percentage of co-morbidities across different age groups of the study participants

Among the study population, 39.5% patients did not have co-existing co-morbidities. Nonetheless, 35.8%, 17.3%, and 3.7% patients had been diagnosed with one, two, three, and four and above co-morbidities, respectively (Fig. 3). Anaemia 21(25.9%), retroviral disease 15(18.3%) and hypertension 13(16.1%) were the most common types of co-morbidities. Conversely, pulmonary embolism, sepsis, acute kidney injury, goitre and gastric ulcer were the least frequent co-morbidities among the study participants (Table 2). When age was taken into consideration, most of the study participants (29.6%) who had co-existing co-morbidities were aged 51 years and above (Fig. 4).

**Table 2:** Types of co-morbidities among patients with cervical cancer

Co-morbidity	Frequency	Percent
Anaemia	21	25.9
Retroviral disease	15	18.5
Hypertension	13	16.1
Hydronephrosis	13	16.1
Deep vein thrombosis	3	3.7
Rheumatoid arthritis	3	3.7
Chronic kidney disease	3	3.7
Type II diabetes mellitus	2	2.5
Acute kidney injury	1	1.2
Pulmonary embolism	1	1.2
Sepsis	1	1.2
Gastric ulcer	1	1.2
Goitre	1	1.2

### Types of regimen used in the management of cervical cancer

Chemoradiation 41(50.6%) comprising of weekly cisplatin and daily radiotherapy was the most widely used treatment regimen in the management of cervical cancer in our setting. Further, hysterectomy and brachytherapy had been used in the management of 15(18.5%) and 11(13.6%) of the patients, respectively. Cisplatin and paclitaxel 9(11.1%) were the most commonly used combination anticancer agents in the treatment of cervical cancer (Table 3).

**Table 3:** Types of regimen used in the management of cervical cancer

Regimen	Frequency	Percent
Chemoradiation (Cisplatin weekly + Radiotherapy)	41	50.6
Hysterectomy	15	18.5
Brachytherapy	11	13.6
Radiotherapy	10	12.3
Cisplatin + Paclitaxel	9	11.1
Carboplatin + Paclitaxel	5	6.2
Cisplatin +Vinorelbine	1	1.2

Granisetron and dexamethasone combination 32(39.5%) was the most commonly used prophylactic antiemetic regimen followed by a combination of ondansetron and dexamethasone 18(22.2%). Conversely, metoclopramide and ondansetron monotherapy were less frequently used in management of chemotherapy-induced emesis among the study subjects (Table 4).

**Table 4:** Types of prophylactic antiemetic regimens used in cervical cancer

Type of antiemetic	Frequency	Percent
Granisetron and dexamethasone	32	39.5
Ondansetron and dexamethasone	18	22.2
Metoclopramide and dexamethasone	4	4.9
Metoclopramide	2	2.5
Ondansetron	1	1.2
No-antiemetics given	24	29.6
Total	81	100.0

The finding of the study showed that paracetamol, morphine, tramadol and codeine were the most commonly used analgesics among the study participants. Nonetheless, significant proportion (37.4%) of cervical cancer patients did not receive any form of pain medication (Table 5).

**Table 5:** Analgesics regimens used in cervical cancer at Kenyatta National Hospital

Type of analgesic	Frequency	Percent
Paracetamol	21	25.9
Morphine	12	14.8
Tramadol	12	14.8
Codeine	11	13.6
Diclofenac	6	7.4
Ibuprofen	5	6.2
meloxicam	3	3.7
Etoricoxib	1	1.2
Analgesic not given	30	37.4

### Prevalence of drug-related problems

A total of 215 DRPs were identified from 76 cervical cancer patients, translating to a prevalence of 93.8% and a mean of 2.65 ± 1.22 DRPs per patient. Adverse drug reactions, drug interactions and the need for additional drug therapy were the most prevalent DRPs, which accounted for 56(69.1%), 38(46.9%) and 32(39.5%) cases, respectively.

In addition, 26(32.1%) patients were non-adherent to their medications, and 16(19.8%) patients received a sub-therapeutic dose of their treatment regimens. Nevertheless, overdosage, improper drug selection, medication use without indication and inappropriate laboratory monitoring accounted for relatively low proportion of drug therapy problems (Table 6).

**Table 6:** Categories of drug related problems

Type of drug related problem	Frequency	Percent
Adverse drug reaction	56	69.1
Drug interaction	38	46.9
Need for additional drug therapy	32	39.5
Non-adherence	26	32.1
Sub-therapeutic dose	16	19.8
Overdosage	15	18.5
Improper drug selection	13	16.1
Medication use without indication	10	12.4
Inappropriate laboratory monitoring	9	11.1

**Fig. 5:**
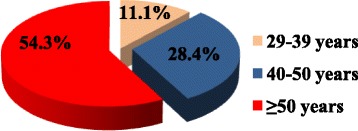
Percentage of drug-related problems based on age group of cervical cancer patients

As illustrated in Fig. 5, most (54.3%) DRPs were found in the 51 years and above age group while the 40–50 years age group accounted for 28.4%. The least proportion of drug-related problems occurred in the 29–39 years age group.

**Fig. 6:**
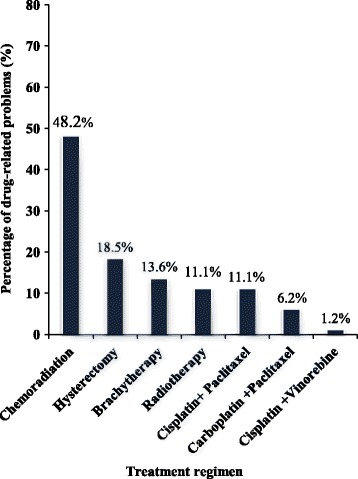
Percentage of drug-related problems across different treatment regimens

As shown in Fig. 6, the predominant proportion of DRPs (48.2%) was identified in patients treated with chemoradiation regimens while 16.1% and 13.6% drug therapy problems were identified in patients who had been managed with radical hysterectomy and brachytherapy, respectively. An equivalent proportion (11.1%) of drug therapy problems were detected in patients treated with radiotherapy and combination of cisplatin and paclitaxel regimens. In contrast, the least proportion of drug therapy problems were identified in patients treated with the combination of carboplatin and paclitaxel and cisplatin and vinorelbine regimens.

**Fig. 7:**
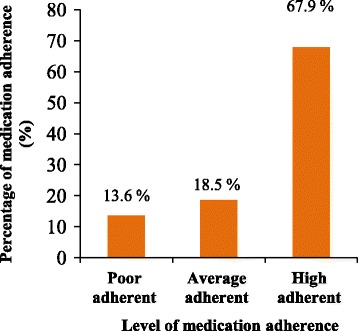
Rate of adherence to medications among cervical cancer patients

According to the eight-item Morisky medication adherence scale, 67.9% of cervical cancer patients were highly adherent, 18.5% of patients had an average level of medication adherence, while 13.6% of patients were poorly adherent to their treatment regimens (Fig. 7).

Forgetfulness 18(69.2%), expensive medications 4(15.4%) and side effects of medications 4(15.4%) were the main reasons for non-adherence to medications in the participants. Long duration of therapy and complicated regimens accounted for equal contribution for medication non-adherence while lack of trust on the efficacy of medications was the least common reason for non-adherence in cervical cancer patients (Table 7).

**Table 7:** Reasons for medications non-adherence among cervical cancer patients (*n* = 26)

Reasons for medications non-adherence	Frequency	Percent
Forgetfulness	18	69.2
Expensive medications	4	15.4
side effects of medications	4	15.4
Long duration of therapy	2	7.7
Complicated regimens	2	7.7
Lack of trust on the efficacy of medications	1	3.8
Others	2	7.7

As indicated in Tables 8, 45 drug-drug interactions were identified among the study participants. Ondansetron and dexamethasone were the most common interacting drugs accounting for 12(26.7%) of the total drug interactions. The other frequently encountered drug interactions were dexamethasone and paclitaxel 4(8.9%), and codeine and morphine 2(4.4%). Each of the other pairs of interacting drugs encountered in this study accounted for approximately 2.2% of the total drug interactions.

**Table 8:** Interacting drugs identified among cervical cancer patients (n = 45)

Severity of the interaction	Interacting drugs	Frequency	Percent
Serious interaction	Codeine + Tramadol	1	2.2
	Metronidazole + Erythromycin	1	2.2
Significant interaction	Amoxicillin +Hydrochlorothiazide	1	2.2
	Zidovudine +cisplatin	1	2.2
	Zidovudine +Cotrimoxazole	1	2.2
	Ceftriaxone + Enoxaparin	1	2.2
	Cisplatin + Gentamicin	1	2.2
	Codeine + Amitryptyline	1	2.2
	Codeine + Morphine	2	4.4
	Dexamethasone + Metronidazole	1	2.2
	Dexamethasone + Tramadol	1	2.2
	Dexamethasone + Paclitaxel	4	8.9
	Diclofenac + Dexamethasone	1	2.2
	Furosemide +Cisplatin	1	2.2
	Nifedipine + Atorvastatin	1	2.2
	Omeprazole + Ranferon	1	2.2
	Ondansetron + Dexamethasone	12	26.7
	Cotrimoxazole +Azithromycin	1	2.2
	Tenofovir + Cisplatin	1	2.2
Minor interaction	Dexamethasone + Amlodipine	1	2.2
	Diclofenac + Enoxaparin	1	2.2
	Eefavirenz + Paclitaxel	1	2.2
	Efavirenz +Tramadol	1	2.2
	Gabapentin + Paracetamol	1	2.2
	Metronidazole + Diclofenac	1	2.2
	Metronidazole + Gentamicin	1	2.2
	Metronidazole + Ibuprofen	1	2.2
	Metronidzole + Paclitaxel	1	2.2
	Nifedipine + Etoricoxib	1	2.2
	Omeprazole + Diazepam	1	2.2

**Fig. 8:**
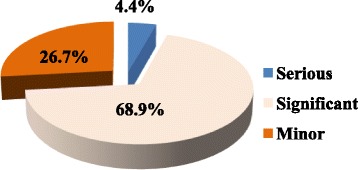
Severity of drug interactions among women with cervical cancer (*n* = 45)

In terms of severity, 68.9% of the drug interactions were significant which required modification or close monitoring of the outcome of the drug interactions. Furthermore, 26.7% of drug interactions were considered as minor interactions. However, 4.4% of drug interactions were serious which necessitate the use of alternative medications in the treatment regimen (Fig. 8).

Of the 166 ADRs identified in this study, the most common were vomiting, nausea, and leucopenia which accounted for 40(49.4%), 24(29.6%), and 18(22.2%) ADRs, respectively. On the other hand, constipation, and thrombocytopenia were the least prevailing ADRs (Table 9).

**Table 9:** Types of adverse drug reactions in cervical cancer patients (*n* = 81)

Types of adverse drug reaction	Frequency	Percent
Vomiting	40	49.4
Nausea	24	29.6
Leucopoenia	18	22.2
Dizziness	13	16.0
Diarrhoea	10	12.3
Abdominal cramp & bloating	8	9.9
Neutropenia	8	9.9
Tinnitus	5	6.2
Low haemoglobin	4	4.9
constipation	3	3.7
thrombocytopenia	1	1.2
Others^a^	15	18.5

^a^Others include hypokalemia, skin rash, oesophageal irritation, bleeding, fatigue, loss of appetite

### Predictors of drug related problems

In the univariable and multivariable binary logistic regression analysis, patients whose cervical cancer was at an advanced stage were 15.4 times (AOR = 15.4, 95% CI = 1.3–185.87, *p* = 0.031) more likely to have DRPs compared to patients with early stage cervical cancer. Hence, stage of cervical cancer was the only predictor of DRPs in cervical cancer patients (Table 10).

**Table 10:** Univariable and multivariable binary logistic regression analysis of predictors of drug related problems

Variable	Univariable analysis		Multivariable analysis	
	COR (95% CI)	*P* value	AOR (95% CI)	*P* value
Age (years)				
29–39	1		1	
40–50	2.6(0.14–46.21)	0.525	3.4(0.13–241)	0.263
≥ 51	1.6(0.15–17.76)	0.689	2.3(0.14–59.22)	0.489
Education				
Illiterate	1		1	
Literate	1.9(0.18–18.81)	0.599	2.5(0.12–48.81)	0.541
Income (USD)				
< 100	1		1	
100–200	0.9(0.09–9.47)	0.938	1.2(0.12–13.52)	0.855
200–500	0.2(0.01–2.08)	0.162	0.1(0.00–1.13)	0.061
Marital status				
Single	1		1	
Married	0.8(0.08–7.23)	0.803	0.9(0.14–5.80)	0.927
Occupation				
Unemployed	1		1	
Employed	0.1(0.01–1.48)	0.096	0.1(0.01–1.41)	1.423
Co-morbidity				
No	1		1	
Yes	1.0(0.16–6.56)	0.982	0.8(0.11–5.11)	0.767
Number of medications				
< 5	1		1	
≥ 5	2.6(0.41–16.53)	0.321	2.5(0.32–21.61)	0.399
Stage of cervical cancer				
Early stage	1		1	
Advanced stage	9.9 (1.45–67.58)	0.019^*^	15.4 (1.3–185.87)	0.031^*^

*COR* Crude odds ratio, *AOR* Adjusted odds ratio, *95% CI* 95% confidence interval, ^*^Statistically significant: *P* value ≤0.05

Patients who had been treated with more than five drugs were 2.9 times (COR = 2.9, 95% CI = 1.10–7.78, *p* = 0.032) more likely to have ADRs as compared to patients treated with less than five medications. In addition, patients with advanced stage disease were 5.9 times (AOR = 5.9, 95% CI = 1.43–24.61, *p* = 0.017) more likely to have ADRs as compared to patients with early stage of cervical cancer. Nonetheless, patients between 40 and 50 years old were 0.1 times (AOR = 0.1, 95% CI = 0.02–0.6, *p* = 0.013) less likely to have ADRs compared to patients with less than 40 years of age (Table 11).

**Table 11:** Univariable and multivariable binary logistic regression analysis of predictors of adverse drug reactions

Variable	Univariable analysis	*P* value	Multivariable analysis	*P* value
	COR (95% CI)		AOR (95% CI)	
Age (years)				
29–39	1		1	
40–50	0.2(0.02–1.45)	0.102	0.1(0.02–0.61)	0.013^*^
≥ 51	0.3(0.03–2.29)	0.227	0.2(0.03–1.2)	0.123
Education				
Illiterate	1		1	
Literate	1.59(0.41–6.26)	0.509	2.4(0.62–9.63)	0.231
Marital status				
Single	1		1	
Married	1.7(0.59–4.99)	0.314	1.7(0.52–5.93)	0.392
Occupation				
Unemployed	1		1	
Employed	0.9(0.08–10.44)	0.925	0.8(0.11–9.04)	0.845
Co-morbidity				
No	1		1	
Yes	0.8(0.30–2.15)	0.668	0.7(0.23–2.31)	0.582
Number of medications				
< 5	1		1	
≥ 5	2.9(1.12–7.78)	0.032^*^	2.9(0.91–9.0)	0.071
Type of cancer				
Adenocarcinoma & Invasive anaplastic carcinoma	1		1	
Squamous cell carcinoma	0.4(0.04–3.09)	0.343	0.1 (0.00–5.42)	0.271
Stage of cervical cancer				
Early stage	1		1	
Advanced stage	4.8(1.37–16.79)	0.014^*^	5.8(1.43–24.61)	0.017^*^

*COR* Crude odds ratio, *AOR* Adjusted odds ratio, *95% CI* 95% confidence interval, ^*^Statistically significant: *P* value ≤0.05

The study revealed that patients with cervical cancer and retroviral disease were 8.8 times (AOR = 8.8, 95% CI = 1.22–68.23, *p* = 0.037) more likely to have drug interactions as compared to cervical cancer patients without concurrent retroviral disease. The other patient factors did not have statistically significant association with drug interactions (Table 12).

**Table 12:** Univariable and multivariable binary logistic regression analysis of predictors of drug interactions

Variable	Univariable analysis	*P* value	Multivariable analysis	*P* value
	COR (95% CI)		AOR (95% CI	
Age (years)				
29–39	1		1	
40–50	1.1(0.22–6.73)	0.957	1.9(0.21–19.11)	0.558
≥ 51	0.2(0.02–1.54)	0.109	0.5 (0.04–6.11)	0.591
Education				
Illiterate	1		1	
Literate	1.1(0.13–10.38)	0.906	0.3(0.04–3.02)	0.318
Marital status				
Single	1		1	
Married	0.6(0.14–2.77)	0.529	1.2 (0.23–6.11)	0.851
Co-morbidity				
No	1		1	
Yes	6.1(0.71–51.61)	0.101	1.2(0.11–16.32)	0.882
Retroviral disease	14.0(2.93–66.72)	0.001^*^	8.8(1.22–68.23)	0.037^*^
Number of medications				
< 5	1		1	
≥ 5	0.45(0.11–1.85)	0.269	0.2(0.03–1.24)	0.081
Type of cancer				
Adenocarcinoma & Invasive anaplastic carcinoma	1		1	
Squamous cell carcinoma	0.3(0.42–1.62)	0.150	0.4(0.02–6.41)	0.723
Stage of cervical cancer				
Early stage	1		1	
Advanced stage	0.6(0.11–3.48)	0.597	1.5(0.21–11.72)	0.651

*COR* Crude odds ratio, *AOR* Adjusted odds ratio, *95% CI* 95% confidence interval, ^*^Statistically significant: *P* value ≤0.05

It was noted that patients treated with more than five drugs were 3.6 times (AOR = 3.6, 95% CI = 1.24–11.23, *p* = 0.026) more likely to have dosing problems as compared to patients treated with less than five medications. Besides, patients who had been managed with cisplatin and paclitaxel regimen were 9.8 times (AOR = 9.8, 95% CI = 1.25–77.81, *p* = 0.030) more likely to have dosing problems than patients who were not using this regimen (Table 13).

**Table 13:** Univariable and multivariable binary logistic regression analysis of predictors of dosing problems

Variable	Univariable analysis	*P*-Value	Multivariable analysis	*P*-value
	COR (95% CI)		AOR (95% CI)	
Age (years)				
29–39	1		1	
40–50	1.7(0.34–8.15)	0.529	2.4(0.32–17.61)	0.412
≥ 51	1.5(0.32–6.39)	0.625	1.7(0.24–11.52)	
Education				
Illiterate	1		1	
Literate	2.8(0.54–14.11)	0.221	4.2(0.60–30.01)	0.161
Marital status				
Single	1		1	
Married	1.6(0.54–4.82)	0.386	1.2(0.42–3.71)	0.761
Co-morbidity				
No	1		1	
Yes	0.4(0.17–1.11)	0.084	0.4(0.11–1.32)	0.125
Number of medications				
< 5	1		1	
≥ 5	3.2(1.15–8.73)	0.026^*^	3.6(1.24–11.23)	0.026^*^
Type of cancer				
Adenocarcinoma & Invasive anaplastic carcinoma	1		1	
Squamous cell carcinoma	1.6(0.29–8.96)	0.586	1.5(0.32–7.43)	0.614
Stage of cervical cancer				
Early stage	1		1	
Advanced stage	2.3(0.58–9.33)	0.231	3.2(0.72–13.62)	0.121
Treatment Regimen				
Cisplatin + Paclitaxel	3.7(0.9–16.5)	0.079	9.8(1.25–77.81)	0.030^*^

*COR* Crude odds ratio, *AOR* Adjusted odds ratio, *95% CI* 95% confidence interval, ^*^Statistically significant: *P* value ≤0.05

## Discussion

The present study revealed that the mean age of the study participants was 53.3 ± 11.6 years, and the predominant portion of the study subjects 47(58.0%) were 51 years and above. This study is fairly comparable with similar studies conducted in India and Tanzania [20, 21]. Late incidence of cervical cancer in the older age may be due to the insidious transformation of the cervical epithelium into cancerous cells by the combined effects of high-risk strains of human papillomavirus (HPV) and other risk factors [22].

Most of the study population had stages IIB (33.3%) and IIIB (28.4%) cervical cancer while stages IA and IB2 were the least prevalent. Likewise, a similar study in India showed that stage IIIB (38%) and stage IIB (35%) were the most common clinical stages found in cervical cancer patients [21]. The high prevalence of locally advanced stage of cervical cancer patients in our setting may be due to inadequate understanding of the early symptoms of cervical cancer and poor habit of early screening. Moreover, since the majority of the patients had a maximum of primary level education, they might have inadequate understanding of the importance for early Pap smear screening leading to the predominance of advanced stage cervical cancer at the time of diagnosis. Most of the patients in stage I were managed using surgical intervention. According to our eligibility criteria, the patients must be on drug or chemotherapy to be included in the study since we are as assessing drug related problems. Hence, majority of the patients in stage I were not eligible to be included in the study. Moreover, advanced radiological imaging techniques such as PET scan were not available in our facility to screen early stage of precancerous lesion in the cervix. That is why cervical cancer patients with stage I were least prevalent in our setting.

The mortality rate after stage IIIB was very high in our setting due to the progression of the disease. Besides, the rate of transfer to more advanced treatment facilities in advanced stage of the disease was very high. Those are the main reasons why cervical cancer patients with stage IV were very limited in our setting.

Most of the study participants (39.5%) did not have co-existing co-morbidities. Nonetheless, 35.8%, 17.3%, and 3.7% patients were diagnosed with one, two, three, and four and above co-morbidities, respectively. In contrast, a similar study in Zimbabwe indicated that majority of the study participants (79.4%) had concurrent co-morbidities [23]. In the present study, the most common co-morbidity was anaemia (25.9%) probably arising from tumour-induced bleeding and iron deficiency secondary to malignancy [24]. This finding is in agreement with an Iranian study in which anaemia was the most common (59.0%) complication among cervical cancer patients [25]. Contrastingly, a study done in Nigeria identified hypertension (29.8%) and diabetes mellitus (27.4%) as the most common co-morbidities in cervical cancer patients [26].

Retroviral disease (18.3%) was the second leading type of co-morbidity in cervical cancer patients. Correspondingly, a cross-sectional study in Zimbabwe showed that 25.6% of the study participants had a retroviral disease [27]. In addition, some studies have shown that a strong association exists between human immunodeficiency virus (HIV) infection and cervical cancer with a high prevalence of high-risk HPV DNA in women with HIV infection [28, 29]. This could probably be due to a weakened immune system secondary to retrovirus infection which puts them at higher risk of HPV infections. Moreover, the retrovirus may augment the oncogenic activities of HPV which predispose the patients to develop cervical cancer [30]. Although thromboembolic disorders are among the top ranked co-morbidities in cervical cancer patients [31], they had relatively low occurrence among the study participants.

Chemoradiation was the most widely used treatment regimen in the management of cervical cancer at KNH accounting for 50.6% of treatment modalities which is higher than in a similar study conducted in Ethiopia (37.6%) [32]. In the present study, cisplatin and paclitaxel (11.1%) were the most commonly used combination anticancer agents in the treatment of cervical cancer. Contrastingly, cisplatin and 5-fluorouracil combination regimen was widely used in a Nigerian study [26].

The study showed that granisetron and dexamethasone combination was the most commonly used prophylactic antiemetic in our setting with a usage frequency of 39.5%, followed by a combination of ondansetron and dexamethasone (22.2%). Serotonin receptor type 3 (5-HT_3_) antagonists such as ondansetron and granisetron are the gold standard treatment protocol for chemotherapy-induced nausea and vomiting due to superior efficacy and better tolerability of side effects as compared to conventional antiemetics [33]. The 5-HT_3_ receptor antagonists are also preferred over dopamine receptor antagonists since they are devoid of extrapyramidal side effects [34]. Previous studies reported that the efficacy of 5-HT_3_-receptor antagonists was augmented with the addition of dexamethasone [35]. Although equivalent doses of different 5-HT_3_-receptor antagonists had comparable efficacy [34], a combination of ondansetron and dexamethasone use was not common in our setting due to drug-drug interaction. This finding corroborated the frequent use of granisetron and dexamethasone combination in our setting which is in line with the standard protocol [35].

The finding of 93.8% prevalence of DRPs in our setting is fairly higher than in a similar Norwegian study (73%) [36]. However, the finding of this study is comparable with a similar study done in Nigeria which showed that the prevalence of DRPs in cervical cancer patients was 89.2% [26]. Besides, a mean of 2.65 ± 1.22 drug therapy problems were identified in the study population which is relatively higher than 2.1 DRPs detected per patient in a study done in Norway [36]. The higher prevalence DRPs in our setting may be due to inadequate understanding of the disease and medications among the patients and absence of local standard treatment protocols for cervical cancer patients.

There was a high preponderance of DRPs in the 51 years and above age group that accounted for 54.3% of the cases. This could probably be due to the high prevalence of co-morbidities in patients 51 years and above (29.6%) and the ageing of the metabolising organs which predispose the patient to DRPs.

Adverse drug reactions (69.1%) and drug interactions (46.9%) were the most prevalent DRPs, a finding that is in agreement with a similar study done in Nigeria [26] but higher than a finding reported by a Singaporean study [37]. The high incidence of ADRs may be attributed to the complexity and immunosuppressive effects of cancer treatment regimens.

Nausea and vomiting were among the top ranking ADRs. These findings are in line with a study done in India in which nausea and vomiting were prevalent among cancer patients treated with anticancer agents [38, 39]. This could probably be linked to the emetogenic potential of cisplatin and paclitaxel and the cytotoxic effects of anticancer agents in the highly proliferating cells of the gastrointestinal tract. Additionally, the higher incidence of nausea and vomiting could be due to poor management of delayed nausea and vomiting secondary to the anticancer agents.

Although morphine, tramadol and codeine were the most commonly used pain medications, only 3.7% of the population had constipation as ADR. In our facility, these pain medications were usually given along with stool softeners and this clinical practice could probably be the main reason why constipation due to these opioids-based pain medications was not a major issue in our setting. Pain control was in line with WHO guideline for pain control in cancer patients and hence we didn’t notice any discrepancies except drug interactions due to the combined use of two opioid analgesics (i.e. codeine and morphine) among 4.4% of the study participants. Pain medications were considered as essential drugs for palliative care treatment in cancer patients in Kenya [40]. Hence, almost all public healthcare facilities offering cancer treatment were universally accessible to these essential drugs. However, controlled pain medications such as opioid analgesics were accessed to cancer patients under supervised prescription by the palliative care specialists. In addition, being controlled drugs those medicines may not be available at the lower level of healthcare facilities.

When age was taken into consideration, elderly patients (age ≥ 51 years) had encountered most (40.7%) of the ADRs. This finding is similar to that reported by Poddar et al. [41] where the incidence of ADRs among geriatric patients was significantly higher than other age groups. This may be due to diminished metabolising capacity and excretory functions in the elderly patients leading to accumulation of drugs in the body and thus increasing the risk of ADRs [42].

Chemoradiation was the most commonly used treatment modality and was also associated with the majority of the ADRs in our setting which is comparable with other studies [43, 44]. Furthermore, the present study revealed that 33.3% and 28.4% patients had stage IIB and IIIB cervical cancer, respectively which were categorised as locally advanced cervical cancer. It has been shown that chemoradiation is the standard treatment of choice in the management of locally advanced cervical cancer due to the overall tolerability of side effects and enhancement of survival [45, 46]. This could probably be the reason why this regimen was widely used in our setting and was therefore associated with the majority of the ADRs.

Due to the complexity of the chemotherapeutic regimens, cancer patients are susceptible to potential drug interactions. Not surprisingly, this study unveiled that 46.9% of cervical cancer patients had potential drug interactions in the treatment regimens. A similar study in Dutch reported 46% prevalence of potential drug interactions among cancer patients [47]. This high prevalence of drug interactions may step up the adverse effects of anticancer agents or lessen the therapeutic outcomes of the treatment regimen. With regard to severity, 68.9% significant drug interactions were detected from the treatment regimens of cervical cancer patients which is slightly higher than a study done in Tehran that reported a prevalence of 59.7% [48]. However, only 4.4% of the drug interactions were identified as serious drug interaction which necessitates use of alternative drug regimens.

Ondansetron and dexamethasone were the most common interacting drugs accounting for 26.7% of the total drug interactions. Previous studies reported that premedication of dexamethasone diminished the efficacy of paclitaxel in breast cancer and ovarian carcinoma [49, 50]. According to the findings of the present study, dexamethasone and paclitaxel accounted for 8.9% of the drug interactions. Thus, it is plausible to assume that the prophylactic use of dexamethasone antiemetic in paclitaxel-based regimens might reduce the antitumor activity of paclitaxel in cervical cancer patients.

A cross-sectional descriptive study conducted in Ethiopia revealed that 69.7% of cervical cancer patients were adherent to their treatment regimens while 30.3% of patients were non-adherent [32]. Similarly, 67.9% of cervical cancer patients were adherent to their treatment regimens in our setting. However, the rate of medication adherence (61.1%) among cervical cancer patients in India was slightly lower than our setting [51]. This could probably be due to the availability of better facilities to strengthen the awareness of the patients about their medications adherence at the Oncology Units of KNH.

Among 26 non-adherent cervical cancer patients, forgetfulness (69.2%), expensive medications (15.4%) and side effects of medications (15.4%) were the main reasons for non-adherence while long duration of therapy and complicated regimens contributed equivalently (7.7%) to medication non-adherence. On the other hand, lack of trust on the efficacy of medicines was the least common reason for non-adherence in cervical cancer patients at KNH. Comparatively, a study from Ethiopia revealed that long duration of therapy, side effects of the medication and expensive medication were among the top-ranking reasons for medication non-adherence in cervical cancer patients [32].

The present study revealed that patients with advanced stage cervical cancer were 15.4 times (AOR = 15.4, 95% CI = 1.3–185.87, *p* = 0.031) more likely to have DRPs as compared to patients with early stage cervical cancer. In addition, patients with advanced stage cervical cancer were 5.9 times (AOR = 5.9, 95% CI = 1.4–24.6, *p* = 0.017) more likely to experience ADRs as compared to patients with early stage disease.

Koh et al. [52] reported that multiple uses of drugs were a significant predictor of the incidence of DRPs. Hence, the higher likelihood of DRPs in the advanced stage cervical cancer may be due to multiple medications secondary to the complexity of the conditions which predispose the patients to DRPs. Likewise, stage of cervical cancer was the only predictor of DRPs in cervical cancer patients. Previous studies in Sweden [53], Malaysia [54], Nigeria [26] and Ethiopia [55] reported that polypharmacy and presence of co-morbidities were positively associated with DRPs. Conversely, our study revealed that number of medications and presence of co-morbidities were not statistically significant predictors of drug related problems.

Patients who had been treated with more than five drugs were more likely to have ADRs and dosing problems and less likely to have inappropriate laboratory monitoring as compared to patients treated with less than five medications. Similarly, previous study in Pakistan showed that polypharmacy was positively associated with ADRs [56]. Moreover, a similar study in Singapore showed that chronic use of five or more drugs was associated with the presence of DRPs [37]. The higher likelihood of having ADRs may plausibly be due to the enhanced pharmacological effects of the drugs secondary to the undesired drug interaction at the level of metabolism and excretion.

In the univariable logistic regression analysis, patients who had been managed with cisplatin and paclitaxel regimen were 9.8 times more likely to have dosing problems. Additionally, cervical cancer patients with the retroviral disease were 8.8 times (AOR = 8.8, 95% CI = 1.2–68, *p* = 0.037) more likely to have drug interactions as compared to patients without concurrent retroviral disease. Conversely, the other sociodemographic factors did not have statistically significant association with drug interactions. The higher likelihood of having drug interactions may plausibly be due to the complexity of drug regimens in the management of both conditions. Previous studies showed that an increased risk of nephrotoxicity due to the combination tenofovir and platinum analogues such as cisplatin particularly in patients with renal insufficiency. Moreover, there was a mounting report of haematological toxicity with a combination of taxane class of anticancer agents such as paclitaxel and zidovudine [57]. Since the majority of cervical cancer patients with retroviral disease were treated with tenofovir and cisplatin-based regimens in our setting, they were a higher risk of having nephrotoxicity due to drug-drug interaction between the anticancer and anti retroviral agents. Hence, having a retroviral disease as co-morbidity in cervical patients might be an important predictor for drug interaction.

## Conclusion

Adverse drug reactions, drug interactions, and need of additional drug therapy were the most common DRPs identified among cervical cancer patients. Nausea and vomiting were the most prevalent ADRs among the study participants. In the multivariable binary logistic regression analysis, advanced stage of cervical cancer and treatment with more than five drugs were significant predictors of ADRs. Likewise, coexisting retroviral disease and treatment with more than five medications were predictors of drug interactions and dosing problems, respectively.

## Abbreviations

ADRs: Adverse Drug Reactions
AOR: Adjusted Odds Ratio
CI: Confidence Interval
COR: Crude Odds Ratio
DRPs: Drug Related Problems
ESMO: European Society for Medical Oncology
GFR: Glomerular Filtration Rate.
NCCN: National Compressive Cancer Network.
USD: United States Dollar.
WHO: World Health Organization
